# The current practice trends in pediatric bone-anchored hearing aids in Canada: a national clinical and surgical practice survey

**DOI:** 10.1186/1916-0216-42-43

**Published:** 2013-07-01

**Authors:** C Carrie Liu, Neil K Chadha, Manohar Bance, Paul Hong

**Affiliations:** 1 Division of Otolaryngology-Head and Neck Surgery, University of Calgary, Calgary, AB, Canada; 2 Division of Pediatric Otolaryngology-Head and Neck Surgery, B.C. Children’s Hospital, University of British Columbia, Vancouver, BC, Canada; 3 Division of Otolaryngology-Head and Neck Surgery, IWK Health Centre, Dalhousie University, Halifax, NS, Canada; 4 School of Human Communication Disorders, Dalhousie University, Halifax, NS, Canada; 5 Foothills Medical Centre, 1403-29 Street NW, South Tower Room 602, Calgary, AB T2N 2T9, Canada

**Keywords:** Bone-anchored hearing aid, BAHA, Surgical practice, Clinical practice, Practice survey, Pediatrics

## Abstract

**Background:**

Since the introduction of bone-anchored hearing aids (BAHAs) in the 1980s, the practices of surgeons who implant these hearing aids have become varied; different indications and surgical techniques are utilized depending on the surgeon and institution. The objective of the current study is to describe the clinical and surgical practices of otolaryngologists in Canada who perform pediatric BAHA operations.

**Methods:**

A detailed practice questionnaire was devised and sent to all members of the Canadian Society of Otolaryngology-Head and Neck Surgery. Those who performed pediatric BAHA surgeries were asked to participate.

**Results:**

Twelve responses were received (response rate of 80%). All of the respondents identified congenital aural atresia to be an indication for pediatric BAHAs. Other indications were chronic otitis externa or media with hearing loss (92%), allergic reactions to conventional hearing aids (75%), congenital fixation or anomaly of ossicular chain (67%), and unilateral deafness (25%). Minor complications, such as skin reactions, were reported in 25% of cases, while major complications were very rare. There was great variability with regards to surgical techinque and post-operative management. The extent of financial support for the BAHA hardware and device also varied between provinces, and even within the same province.

**Conclusion:**

There is a lack of general consensus regarding pediatric BAHA surgeries in Canada. With such a small community of otolaryngologists performing this procedure, we are hopeful that this survey can serve as an impetus for a national collaboration to establish a set of general management principles and inspire multi-site research ventures.

## Introduction

The application of osseointegration for the purpose of hearing rehabilitation was first introduced in 1977 [1]. Now, commonly referred to as bone-anchored hearing aid (BAHA), this technique supplanted bone conduction hearing aids, leading to a delivery of more robust and higher quality sounds [2]. Such a system allows for sound to be transmitted directly to the cochlea through the cranium, circumventing any external or middle ear anomaly or pathology. Numerous studies have confirmed the benefits of the BAHA system, or a very similar system from Oticon Medical (Askim, Sweden), the Ponto®, in terms of audiological outcome, aesthetics, and health-related quality of life [2-6].

Since their commercial introduction in 1987, BAHAs have become a common treatment option for patients with conductive hearing loss who are either unsuitable for or have failed conventional hearing aids [1-3,7]. Originally, their use was limited to those with chronic otitis media [7], yet with time the indications have grown to encompass numerous conditions, including congenital ear anomalies, chronic otitis externa or media, and single-sided deafness [2-4,6-10]. Along with the escalation in indications, there has been a divergence in the clinical and operative practices of otolaryngologists who perform pediatric BAHA surgeries. Depending on the surgeon and health centre experiences, different indications and techniques are utilized, resulting in variable practices [7].

The present study describes the clinical and surgical practices of otolaryngologists in Canada who perform pediatric BAHA operations. Elucidating the trends and variations in surgeon preferences and practices in one country may inspire a national collaboration to establish a set of general management principles with regards to pediatric BAHAs. It may also serve as an impetus for multi-site research ventures and information for policy and decision makers.

## Methods

A practice survey questionnaire was devised by the authors based on a review of the literature as well as from acquired experiences. The survey consisted of 39 questions divided into general practice and surgical practice sections (Additional file 1: Appendix A). The general practice section comprised of questions regarding demographic information, including province of practice and completion and type of training. Respondents were then asked about their indications for pediatric BAHA surgeries as well as their use of the BAHA softband. Finally, there were questions regarding the funding of unilateral and bilateral BAHAs, replacement BAHAs, the BAHA softband, and perioperative complications.

The surgical practice section of the questionnaire inquired about the placement of the BAHA implant in microtia and non-microtia children, incision type, and the use of dermatomes and bony augmentation. There were also questions regarding the age of BAHA implantation, indications for one- and two-staged procedures, length of time in between stages, the placement/use of sleeper implants, and patient follow-up.

The questionnaire was sent via email to all members of the Canadian Society of Otolaryngology – Head and Neck Surgery (CSO-HNS). Only those who performed pediatric BAHAs were asked to respond. Two reminder emails were sent over the course of three months.

Given that this study is not experimental in nature and did not involve any patients or animals, exemption was obtained from the local Institutional Review Board.

## Results

A total of 12 responses were received. Specifically, a response rate of 80% was obtained since there were 15 otolaryngologists performing pediatric BAHA operations at the time of the survey in Canada [deduced from contacting the BAHA manufacturers (Cochlear and Oticon) and based on the pediatric BAHA surgeons already known to the authors in different regions].

### General practice

Table 1 shows the demographic data of the respondents. There were no representations from the provinces of Saskatchewan, Manitoba and New Brunswick, and the Yukon, Nunavut and Northwest Territories. This was expected since these regions did not have a pediatric BAHA program at the time of the survey. Eleven respondents worked in academic institutions and 10 have undergone fellowship training. Four were fellowship trained in pediatric otolaryngology and six in otology/neurotology. The average number of pediatric BAHAs implanted each year per surgeon was 6.4 (range 1–20).

**Table 1:** Demographic data of survey respondents

	**Respondents**	
	**Number**	**Percent**
Province		
British Columbia	1	8
Alberta	2	17
Ontario	3	25
Quebec	2	17
Nova Scotia	3	25
Newfoundland and Labrador	1	8
Academic institution		
Yes	11	92
No	1	8
Fellowship training		
Yes	10	83
Otology/Neurotology	6	60
Pediatric Otolaryngology	4	40
No	1	8
Not specified	1	8

Table 2 summarizes the clinical practices of the respondents. All respondents identified congenital aural atresia to be an indication for performing pediatric BAHAs. Other indications were chronic otitis externa or media with hearing loss (92%), allergic reactions to conventional hearing aids (75%), congenital fixation or anomaly of the ossicular chain (67%), and unilateral deafness (25%). Otologists who completed the survey have a median of 4 indications (range 4 to 6), while pediatric otolaryngologists have a median of 3 indications (range 2 to 5). None of the Canadian pediatric otolaryngologists stated unilateral deafness as an indication for pediatric BAHAs.

**Table 2:** Summary of clinical practices

	**Respondents**	
	**Number**	**Percent**
Would you routinely offer a BAHA for congenital unilateral conductive hearing loss in children?		
Yes	2	17
No	10	83
Indications for pediatric BAHAs		
Congenital atresia of ear canal	12	100
Chronic otitis externa or media with hearing loss	11	92
Allergic reactions to standard hearing aids	9	75
Congenital fixation or anomaly of ossicular chain	8	67
Unilateral deafness	3	25
Trisomy 21	5	42
Treatment of external auditory canal atresia		
Primarily canalplasty	1	8
Primarily BAHA	9	75
50% canalplasty, 50% BAHA	1	8
Complete canal atresia - BAHA, partial canal stenosis - canalplasty	1	8
Have you ever performed bilateral BAHAs in children? How many?		
Yes - 1-3	4	33
Yes - 5	1	8
Yes - 10-15	1	8
No	6	50
Do you routinely use the BAHA softband in children if indicated?		
Yes	11	92
No	1	8
At what age would you fit the BAHA softband in bilateral conductive hearing loss? (months)		
2	1	8
4-5	2	17
6	4	33
12	4	33
No response	1	8
Do you have a dedicated BAHA audiologist at your institution?		
Yes	11	92
No	1	8
Have you ever implanted other devices? (e.g., Vibrant Soundbridge) for pediatric conductive hearing loss?		
Yes	1	8
No	11	92

In the management of external auditory canal atresia, nine respondents (75%) primarily performed BAHAs. For three of these respondents, the percentage of children treated with BAHAs and canalplasties were 75-80% and 20-25%, respectively. One surgeon reported performing primarily canalplasties, and BAHAs were reported to be reserved only for patients who had unsuccessful canalplasty outcomes. All respondents with subspecialty training in pediatric otolaryngology used BAHAs as the primary treatment for external auditory canal atresia. For those with training in otology/neurotology, BAHAs were used as primary treatment for three (50%) respondents.

Costs associated with bilateral BAHAs were reportedly covered in the practice areas of seven respondents (58%, Table 3). Of these, four respondents reported full cost coverage for all BAHA-related components. One respondent did not specify and two reported partial coverage. Three respondents (25%) reported full coverage for the first BAHA only and two (17%) reported only partial coverage for the first BAHA. Out of the 12 respondents, five reported the availability of government funding for replacement BAHAs.

**Table 3:** Funding for costs associated with bilateral BAHAs

**Province**	**Funding, partial (P)/full (F)**	**If partial – portions covered**	**Alternative funding**	**Replacement BAHA coverage**	Coverage of BAHA softband *
BC	No	Partial funding for one BAHA - implant, procedure, not processor	Provincial program	No	Yes
AB	Yes (F)	--	--	Yes	Yes
AB	Yes (P)	Procedure	Charity foundation	Yes	Yes
ON	Yes (F)	--	--	Yes	No
ON	Yes (F)	--	--	No	No
ON	No	Full coverage for one BAHA	Private insurance	No	Yes
QC	No	Partial funding for one BAHA - implant, processor	None	No	No
QC	Yes (P)	Procedure	Charity foundation	No	No
NS	No	Full coverage for one BAHA	Private insurance	No	Yes
NS	Yes (−)	--	--	Yes	Yes
NS	Yes (F)	--	--	Yes	Yes
NL	No	Full coverage for one BAHA	None	No	Yes

-- No response.

* Coverage of BAHA softband in the absence of an implanted BAHA.

BC - British Columbia, AB - Alberta, ON - Ontario, QC - Quebec, NS - Nova Scotia, NL - Newfoundland and Labrador.

Six surgeons (50%) have performed bilateral BAHAs in children, ranging from one to 15 cases each. The main indications included bilateral external auditory canal atresia and inability to wear conventional hearing aids, bilaterally. Costs associated with bilateral BAHAs were covered (either fully or partially) in the practice regions of five of the six surgeons (83%) who have performed this procedure in children. There was no coverage for bilateral BAHAs in the practice regions of four of the six surgeons (67%) who have not performed this procedure in children. Eleven out of 12 respondents (92%) reported that they routinely use the BAHA softband in young children when indicated. The BAHA softband was funded in most regions.

Minor complications, which mainly included skin reactions, cellulitis and soft-tissue infections, were reported to affect 25% of pediatric patients. Major complications (loss of implant, hematoma and complete skin overgrowth) were reported in less than 5%.

### Surgical practice

Table 4 summarizes the surgical practices of those who responded to the survey. Eight respondents gave specific measurements for placement of the BAHA implant. The most commonly reported distance was 5 to 5.5 cm posterosuperior from the ear canal opening (or assumed opening in atretic ears), and 42% reported altering the implant site in children with microtia compared to children with normal auricles.

**Table 4:** Summary of surgical practices

	**Respondents**	
	**Number**	**Percent**
For patients with microtia, do you consult the reconstructive ear surgeon for advice on implant location?		
Yes	9	75
No	3	25
Location of implant for non-microtia children		
3.5-4.5 cm posterosuperior to EAC	2	17
5.0-5.5 cm posterosuperior to EAC	5	42
6.5 cm posterosuperior to EAC	1	8
Posterosuperior to EAC, measurement not specified	2	17
No answer	2	17
Location of implant for microtia children		
Same as for non-microtia children	4	33
Further posteriorly or superiorly	5	42
Variable	1	3
No answer	2	17
What are your indications for 2-staged procedures in children?		
Bone thickness/condition and age	6	50
Bone thickness/condition only	2	17
Age only	2	17
Single stage procedures only	1	8
No answer	1	8
Incision type		
Linear	5	42
U-shaped	7	58
Dermatome use		
Yes	6	50
No	6	50
Do you perform pre-operative imaging to assess the thickness or quality of the bone?		
Yes	3	25
No	8	67
No answer	1	3
Do you use Gortex® or other bony augmentation?		
Yes	3	25
No	9	75
What is the earliest age at which you would typically perform the BAHA surgery? (years)		
1.5	1	8
3	3	25
4	5	42
5-6	3	25
What are your indications for a 3 mm versus 4 mm fixture?		
Bone thickness	9	75
4 mm preferred, 3 mm fixtures used if there is inadequate bone thickness	5/9	56
4 mm fixtures used in 80% of cases, 3 mm in 20% of cases	2/9	22
3 mm fixtures typically used in young children	2/9	22
4 mm fixtures regardless of bone thickness	1	8
Age	1	8
No answer	1	8
Length of time between 1^st^ and 2^nd^ stage? (months)		
3	1	8
4-6	4	33
3 for children >6yo, 6 for children <6yo	1	8
6-12 depending on age and bone quality	1	8
No timeline specified but depends on age and bone thickness/quality	1	8
Only perform single-stage surgeries	1	8
No answer	3	25
Do you use of a mastoid dressing after the 1^st^ stage procedure?		
Yes	9	75
No	2	17
No answer	1	8
Do you routinely admit children after stage 1 or 2 procedures?		
Yes	6	50
No	5	42
No answer	1	8
Do you routinely place a sleeper implant during a pediatric case?		
Yes	7	58
No	4	33
No answer	1	8
If yes to the above question, how often have you had to use the sleeper implant?		
Never	4/7	57
Once	1/7	14
Twice	1/7	14
4% of cases	1/7	14
Do you perform soft-tissue reduction during the 1^st^ or 2^nd^ stage?		
1^st^ stage	2	17
2^nd^ stage	8	67
No answer	2	17
If you perform 2-stage operations, how many days after the 2^nd^ stage procedure do you place the sound processor on the abutment?		
7-14	2/9	22
14-30	4/9	44
60-90	3/9	33
How often do you see pediatric BAHA patients have a successful operation?		
Every 1 month, then every 12 months	1	8
Every 3 months, then every 6 months	1	8
Every 6–12 months	9	75
Every 6 months for 24 months, then every 12 months	1	8

*EAC* - external auditory canal.

Incision type varied (42% linear; 58% U-shaped) and 25% routinely performed pre-operative imaging to assess the thickness and quality of the cranial bone. One quarter of the respondents reported using Gortex® or other forms of bone augmentation in select cases.

Nine respondents (75%) stated that bone thickness was the main determinant of the length of fixture used (3 mm versus 4 mm). Of these nine responses, five stated that they would always attempt to use a 4 mm fixture unless the bone thickness is incompatible, in which case a 3 mm fixture will then be implanted. For one surgeon, 4 mm fixtures were always used regardless of bone thickness. Finally, one listed age, rather than bone thickness, as the main determinant of fixture selection.

The wait time between the first and second stage of the procedure ranged from 3 to 12 months. Of those who provided specific timelines, the majority (86%) waited from 3 to 6 months. Time elapsed between second stage and the placement of the sound processor ranged from 7 to 90 days. Seven out of 11 (64%) surgeons routinely placed a sleeper (back-up) implant and three have had to use these back-up implants in the past.

The mean age for performing BAHA surgery was 4 years (range 1.5 to 6 years).

## Discussion

Our findings show that there is variability in the clinical and surgical practices of otolaryngologists who perform pediatric BAHA surgeries in Canada. This is consistent with the divergence in practice that is reported in literature [7]. There is inconsistency among respondents with regards to using unilateral hearing loss and trisomy 21 as indications for BAHA implantation. The practice of bilateral BAHA implantation as well as the minimum acceptable age for implantation also varies. Finally, there is significant regional variation in funding for the BAHA surgery, implant, and processor.

Overall, the least commonly reported indication for BAHA implantation was unilateral hearing loss. The utility of BAHAs in these patients is mainly to establish binaural hearing [11,12]. The reported audiological benefits, however, have been variable in this particular situation, especially in the pediatric population [11,13-15]. In their study of children and adolescents with unilateral hearing loss, Priwin et al [14]. did not find any improvements in hearing thresholds or sound localization with the BAHA. They did, however, note an improvement in speech recognition, especially in noisy environments. This correlates with other studies in which participants have reported using the BAHA mainly in classrooms [11]. Despite the inconsistencies in reported audiological benefits, BAHAs in children with unilateral hearing loss have been found to have a positive impact on quality of life with high rates of user compliance [14-16]. This may suggest that children experience a subjective benefit even if audiological measurements do not always correspond. Twenty-five percent of our respondents listed unilateral hearing loss as an indication for BAHA implantation, and only 17% routinely offered this treatment to children with congenital unilateral hearing loss. This indicates that the majority has not accepted BAHAs as a beneficial intervention for this cohort of children, which seems appropriate at the current level of evidence.

Trisomy 21 was another uncommon indication according to our respondents (Table 2). Children with trisomy 21 have eustachian tube dysfunction, leading to chronic otitis media with effusion and often, conductive hearing loss [17]. Bone anchored hearing aids have been shown to be beneficial in these children when other methods of re-establishing hearing (ventilation tubes and conventional hearing aids) are unsuccessful [17,18]. Despite these results, there appears to be a trend of underutilization of BAHAs in some syndromic children [6]. The lower proportion of respondents who use trisomy 21 as an indication for BAHA implantation may be interpreted to be a reflection of this trend. This may be due to a lack of awareness regarding the benefits of BAHAs in children with trisomy 21 [6]. Increased awareness and education can therefore be helpful in encouraging otolaryngologists to consider BAHAs as a viable option for hearing restoration in this population in some situations.

The benefits of bilateral BAHA implantation have been debated in the literature [19]. Due to the small attenuation of vibrations in the skull, it has been argued that one BAHA can also stimulate the cochlea on the opposite side [10,20]. There have been studies, however, which report improved audiological outcomes and quality of life in children fitted with bilateral BAHAs [16,21,22], indicating that there may be a role for this intervention in some children. Fifty percent of our respondents have performed bilateral BAHAs in children. Interestingly, most surgeons (83%) who have performed bilateral BAHAs practice in an area where the procedure is partially or fully funded, whereas most of those who have not performed this procedure (67%) work in areas where bilateral BAHAs are not funded. It is possible that in the small population of children who may benefit from this intervention, bilateral BAHAs are underused as a result of funding limitations. A similar trend is expected in many other countries since health care funding is becoming more scarce. As we will discuss later on, there is a need for more comprehensive coverage for the BAHA procedure and related costs. We encourage otolaryngologists who practice in regions with funding limitations to advocate for more adequate coverage, especially in situations where the lack of funding may be the obstacle to an intervention that is known to lead to improved outcomes, such as bilateral BAHA implantation in select cases [19].

There is no consensus on the ideal age for BAHA implantation in children [6,9]. Achieving optimal hearing earlier in life best facilitates normal speech and language development [23,24]. Younger age at the time of intervention, however, is associated with an increased risk of osseointegration failure [3,9]. This is most likely due to thinner temporal bones [9] as well as the higher water and lower mineral content associated with younger skulls [25]. Other factors that may contribute to failure in younger children include an increased risk of trauma and a decreased ability to care for the implant site [3,25]. The trend reported in literature is to implant BAHAs in children older than 4 years of age [7]. Others state that children older than 3 years should have adequate bone thickness, and therefore would make suitable BAHA candidates [1]. Our results show that 4 years is the most commonly accepted minimum age for BAHA implantation (42%), followed by 3 years (25%) and 5–6 years (25%). The BAHA softband is routinely fitted for children younger than this for 92% of respondents. Our results imply that there are some children in Canada who are being delayed the BAHA operation secondary to surgeon practices/preferences. By elucidating the fact that the majority of surgeons are successfully performing this procedure in children at age 3–4 years, perhaps those waiting until age 5–6 years would consider implanting the BAHA at an earlier age. This is important since the BAHA processor with implanted abutment has been shown to yield better audiologic results compared to the BAHA headband alone [26]. Therefore, children would benefit from earlier implantation as soon as they are physiologically/anatomically ready to receive the implant.

Similar to how age and bone thickness dictate the timing of the BAHA surgery, these factors were also reported to determine the details of the procedure. Half of the respondents stated that young age and thin temporal bone are indications for two-staged procedure. Few (17%) only considered bone thickness and another 17% only considered age. This is consistent with the literature, where surgeons have reported age and bone thickness to be the major determinants in deciding between a one- or two-staged operation [3,13,25]. Some have reported performing two-staged procedure in children with less than 2.5 mm of bone thickness [25]. Similarly, there is support for one-staged procedure in older children whose bone thickness would allow for the implantation of a 4 mm fixture [3].

One of the most striking results seen in our survey is the variability in the availability and extent of funding for the BAHA surgery, implant, and processor. In some cases, this inconsistency in funding was found to exist even within the same province. Furthermore, there was a lack of reliable funding for post-operative care (e.g., for replacement BAHA sound processors). Others in the past have also recognized the need for more comprehensive funding programs in Canada [8]. Interestingly, one of the challenges encountered is that the BAHA is often considered to be a surgical procedure and not a hearing aid. This distinction can be important since some private health insurance companies provide coverage for hearing aids but not for surgical procedures (or costs related to surgeries). Subsequently, a major challenge identified by parents, which may contribute to a delayed provision of hearing aids, is having to self-fund the costs [27,28]. We hope that by highlighting this discrepancy and possible inadequacy in funding, we are able to encourage a concerted effort among this small group of practitioners to advocate for more extensive coverage for BAHAs for the indicated cases. Obviously, the situations can be quite different in countries such as the United States, where the healthcare funding does not come from a single payer. Yet, many developed nations will have similar funding issues as the ones identified in our survey. Also, even places like the United States may soon have to deal with “rationing” health care resources due to rising costs.

The limitation of the present study is evident in the survey format, which implies reporting and recall bias. A detailed chart review of all pediatric BAHA patients may have avoided these biases but it was not practical. Also, the low sample size can be considered a limitation. However, this small sample size reflects the small group of surgeons in Canada who perform this procedure in children. This is in keeping with the overall small number of children that require this operation and the vast geographical spread with concentrated populations at urban settings. As well, several Provinces and Territories do not have enough population to support their own pediatric BAHA program, which is evident in our survey results. Other countries or regions may have similar findings with a small number of surgeons performing pediatric BAHA operations.

## Conclusion

There is some agreement among otolaryngologists in Canada who perform pediatric BAHA surgeries. Specifically, the predominant indications for BAHA implantation are clear. The remainder of our findings showed a varied set of general and surgical practices among those who perform this procedure in children. It also highlights the discrepancy in government funding across provinces for unilateral and bilateral BAHAs, as well as the BAHA softband.

Given this variation, a national forum would be beneficial to allow for the discussion of the inconsistent practices and contentious issues in BAHA implantation. Specifically, such a forum should establish the types of patients/situations in which unilateral hearing loss would be an indication for BAHA implantation, or when bilateral BAHA implantation should be considered. Furthermore, awareness needs to be raised for surgeons caring for children with Trisomy 21 as to when BAHA implantation may become a viable option. Establishing a set of general practice guidelines in pediatric BAHA surgeries will help ensure that care of these children is as evidence-based as possible. We also hope that this study has raised questions that will inspire multi-institutional research collaborations.

Finally, we encourage otolaryngologists performing BAHA surgeries to become familiar with the various sources of funding available in their regions and to advocate for more comprehensive coverage if a need is identified, both individually at the local level and collectively at the provincial and national levels.

## Competing interests

The authors have no financial disclosure or conflicts of interest.

This material has never been published and is not currently under evaluation in any other peer-reviewed publication.

## Authors’ contributions

CCL participated in the design of the survey, acquiring, analyzing, and interpreting the data, and drafting, revising, and approving the final manuscript. NKC participated in the conception and design of the study, analyzing and interpreting the data, and revising and approving the final manuscript. MB participated in the conception and design of the study, analyzing and interpreting the data, and revising and approving the final manuscript. PH participated in the conception and design of the study, acquiring, analyzing, and interpreting the data, and revising and approving the final manuscript.

## Supplementary Material

Additional file 1 **Appendix A – The Canadian Pediatric Bone-Anchored Hearing Aid Clinical and Surgical Practice Questionnaire.** This is the survey used in this study, consisting of 39 questions divided into general practice and surgical practice sections. Click here for file
