# Supplementary material for: The current practice trends in pediatric bone-anchored hearing aids in Canada: a national clinical and surgical practice survey
Source: J Otolaryngol Head Neck Surg. 2013 Jul 1;42(1):43. doi: 10.1186/1916-0216-42-43 (PMC3702399; doi:10.1186/1916-0216-42-43)
Supplement: Additional file 1 — Appendix A – The Canadian Pediatric Bone-Anchored Hearing Aid Clinical and Surgical Practice Questionnaire. This is the survey used in this study, consisting of 39 questions divided into general practice and surgical practice sections. [file 1916-0216-42-43-S1.pdf]

**The Canadian Pediatric Bone-Anchored Hearing Aid Clinical and Surgical Practice Questionnaire**

General Practice Questions

1. Is your practice at an academic (i.e., University-affiliated) institution?
2. In which province are you currently practicing?
3. Are you fellowship trained?
    a. If yes, what type of fellowship did you complete?
    b. What year did you complete your fellowship training?
4. On average, approximately how many pediatric BAHAs do you perform each year?
5. Approximately how many pediatric BAHAs have you performed in your career?
6. Would you routinely offer a BAHA for congenital unilateral conductive hearing loss in children?
7. What are your indications for pediatric BAHAs?
8. At your institution, is external auditory canal atresia primarily treated with surgery (canalplasty) or with BAHA (or other) implants? Please estimate the percentage treated with each option.
9. Have you ever performed bilateral BAHAs in children?
10. What are your indications for bilateral BAHAs?
11. Are costs associated with bilateral BAHAs covered by your hospital or government?
12. If there is coverage, then does your province offer partial or full coverage of the BAHA procedure, implant hardware, and external sound processor costs?
    a. If there is partial coverage, then which portions are covered?
    b. Are there alternative sources of funding available?
13. Are the costs associated with postoperative care (e.g., replacement BAHAs) covered by the government?
14. Do you routinely use the BAHA softband in children if indicated?
15. Will your province cover the BAHA softband if there is no implanted BAHA?
16. At what age would you fit the BAHA softband in bilateral conductive loss?
17. Do you have a dedicated BAHA audiologist (i.e., an audiologist who manages most of the BAHA patients) at your institution?
18. Have you ever implanted other devices, such as the Vibrant Soundbridge®, for pediatric conductive hearing loss?
19. Approximately what percentage of your patients has had minor complications? Please list them.
20. Approximately what percentage of your patients has had major complications? Please list them.

Surgical Practice Questions

1. For patients with microtia, do you consult the reconstructive ear surgeon for advice on implant location?
2. Where do you usually place the implant for non-microtia children?
3. Where do you usually place the implant for microtia children?
4. What are your indications for 2-staged procedures in children (e.g., age limit, radiation)?

5. What type of incision do you use? (U-shaped, straight-line, others)
6. Do you use a dermatome?
7. Do you perform any pre-operative imaging to assess the thickness or quality of the skull bone?
8. What is the earliest age at which you would typically perform the BAHA surgery?
9. What are your indications for a 3 mm vs 4 mm fixture, and what percentage of each would you use in children?
10. Do you use Gortex® or other bony augmentation?
11. For 2-staged procedures, how long do you usually wait between surgeries? Please stratify by age if necessary.
12. Do you use a mastoid dressing after the 1st stage procedure?
13. Do you routinely admit children after stage 1 or 2 procedures?
14. Do you routinely place a sleeper implant (back up) during a pediatric case?
15. How often have you had to use the sleeper implant?
16. Do you perform soft-tissue reduction during the 1st or the 2nd stage?
17. If you perform 2-staged operations, how many days after the 2nd stage procedure do you place the sound processor on the abutment?
18. How often do you see your pediatric BAHA patients after a successful operation (e.g., every 6 months)?
19. Do you use both Cochlear™ and Oticon Medical™ implants and processors?
    a. If you use both, what percentage are Cochlear™ and what percentage are Oticon Medical™?
    b. How is the decision made when choosing between Cochlear™ and Oticon Medical™ implants and processors? For example, is the decision made by the surgeon, the patient, the audiologist, or the institution?
